# Supplementary material for: Single-Cell RNA Sequencing and Microarray Analysis Reveal the Role of Lipid-Metabolism-Related Genes and Cellular Immune Infiltration in Pre-Eclampsia and Identify Novel Biomarkers for Pre-Eclampsia
Source: Biomedicines. 2023 Aug 21;11(8):2328. doi: 10.3390/biomedicines11082328 (PMC10452287; doi:10.3390/biomedicines11082328)
Supplement: Supplementary file 1 [file biomedicines-11-02328-s001.zip › Supplementary Figures.pdf]

Supplementary Figures

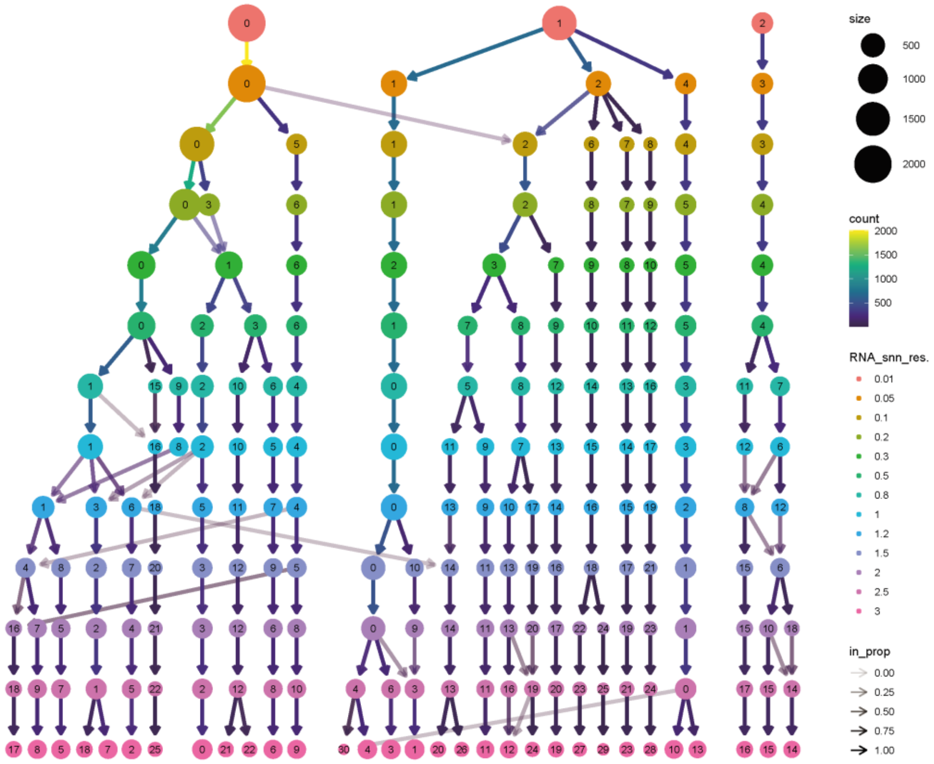

Figure S1. Cluster tree of this analysis and resolution was set as 1.2

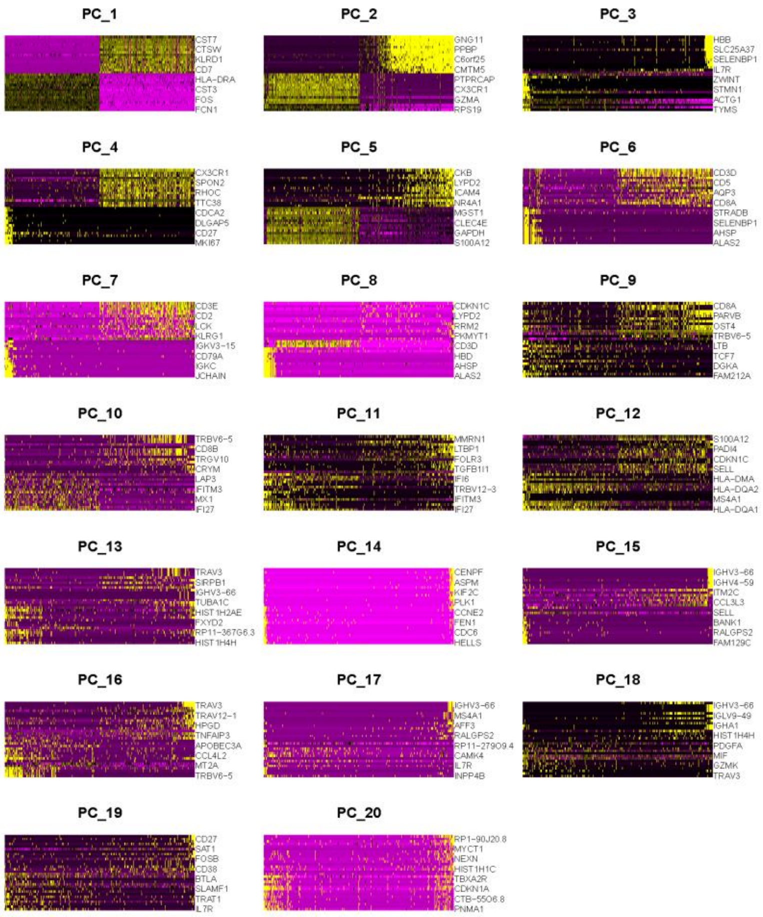

Figure S2. The heatmap shows the expression of the top 8 marker genes in PC

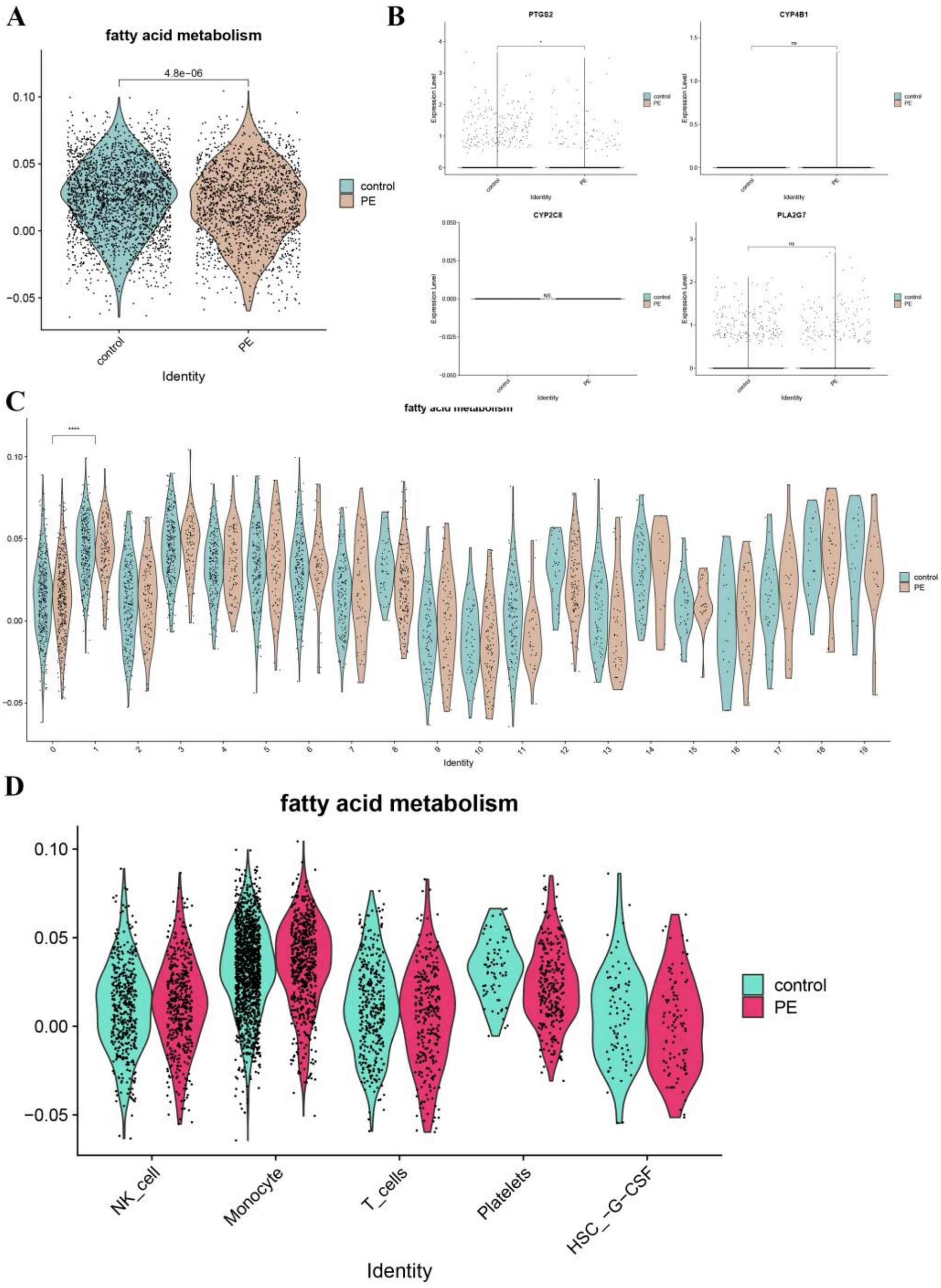

Figure S3. Difference analysis of fatty acid metabolism-related genes between the PE and control groups. (**A**) The differential expression of fatty acid metabolism-related genes between the PE and control groups. (**B**) The differential expression of PTGS2, PLA2G7, CYP2C8, and CYP4B1. (**C**) The differential expression of fatty acid metabolism-related genes between the PE and control groups across 20 clusters. (**D**) The differential expression of fatty acid metabolism-related genes between the PE and control groups across 5 cell types

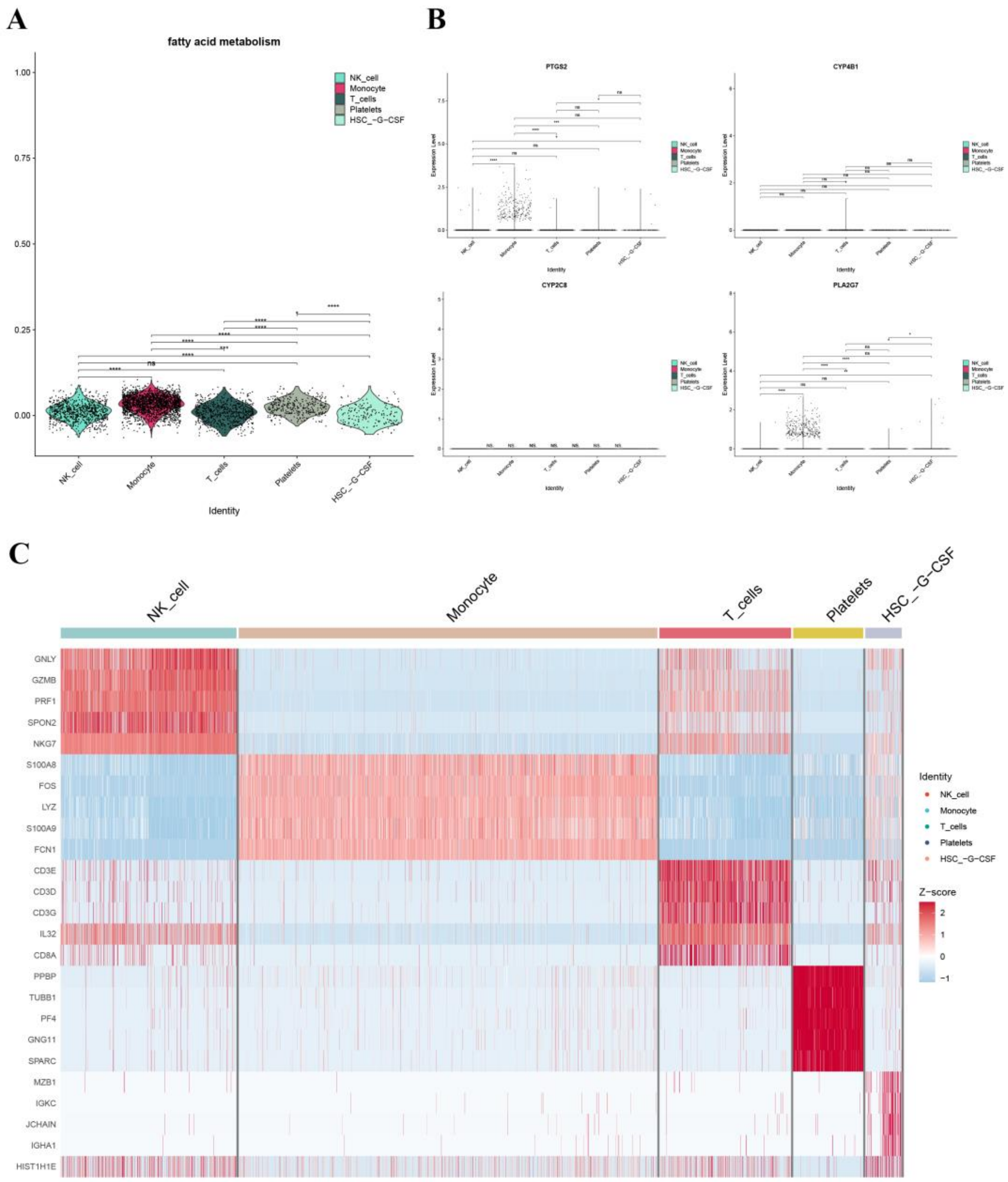

Figure S4. (**A**) The violin plot illustrates the expression of fatty acid metabolism-related genes in each cell type. (**B**) The violin plot illustrates the expression of PTGS2, PLA2G7, CYP2C8, and CYP4B1 in each cell type. (**C**) The heatmap shows the expression of the top 5 marker genes in different cell clusters.

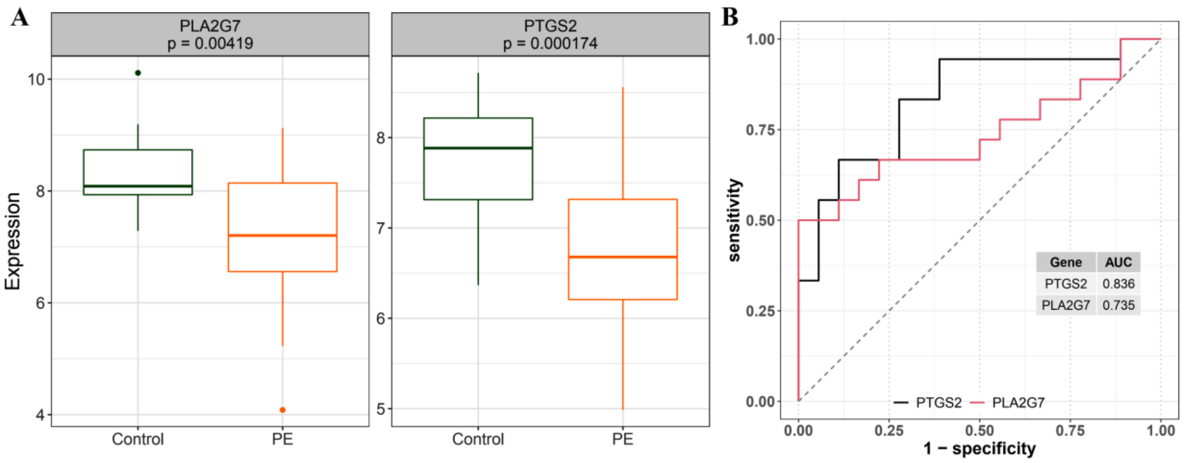

Figure S5. (**A**) Expression of hub genes (**B**) ROC of hub genes.

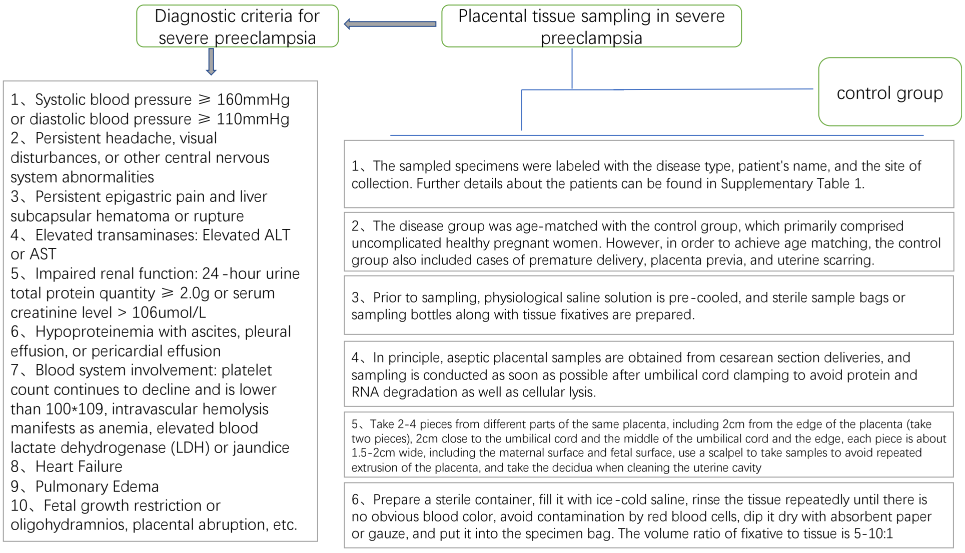

Figure S6. Inclusion criteria for sample collection.

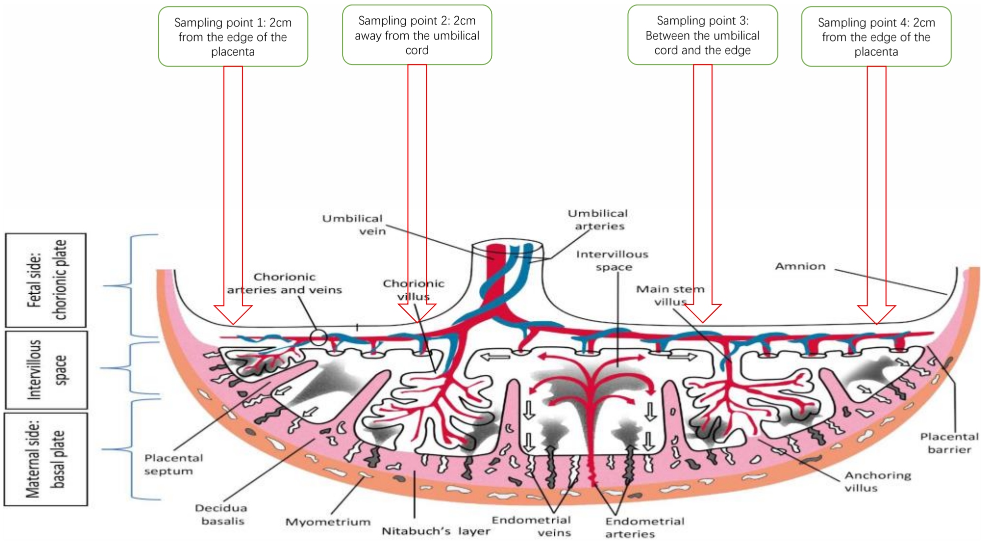

Figure S7. The location where the sample was taken during sample collection.
